# Neuronal Cell‐Type Susceptibility in CA1 in 5xFAD Mice

**DOI:** 10.1002/alz70855_105879

**Published:** 2025-12-24

**Authors:** Maricarmen Pachicano, Shrey Mehta, Angela Hurtado, Michael S Bienkowski

**Affiliations:** ^1^ Stevens Neuroimaging and Informatics Institute, Keck School of Medicine, University of Southern California, Los Angeles, CA, USA

## Abstract

**Background:**

Identification of cell‐type vulnerability in Alzheimer's Disease (AD) is critical to the clinical development of targeted treatments. Neurodegeneration of CA1 is significantly implicated in AD, but it is unknown if specific CA1 cell types are susceptible to AD neurodegeneration. In the 5xFAD mouse model, significant degenerative changes occur within the CA1 by 4‐6 months of age. Our previous work creating the mouse Hippocampus Gene Expression Atlas (HGEA) has defined cell‐type specific gene markers that can identify cell types within CA1. Within CA1, 4 relatively discrete layers of gene expression define cell type organization. Quantification of CA1 cells identified by these gene markers can be utilized to determine CA1 cell type susceptibility across disease timepoints.

**Method:**

To label CA1 gene expression patterns, we used RNAscope single molecule fluorescent in situ hybridization (smFISH) to label and quantify individual RNA transcript levels within CA1 cell types. 5xFAD and wildtype (WT) mice (6, 12, and 14 mo.) were perfused, dissected, and coronally sectioned into 20μm thick coronal sections. CA1 tissue sections were hybridized with probes designed to target CA1 gene expression markers using RNAscope Multiplex Fluorescent V2 kit (Advanced Cell Diagnostics). Tissue sections were counterstained for DAPI cytoarchitecture, imaged at 40X with a spinning disk confocal microscope, and quantification was performed using QuPath (Quantitative Pathology and Bioimage Analysis) cell segmentation and SCAMPR analysis software.

**Result:**

RNAscope labeling produced robust datasets enabling quantification of 100,000+ single RNA transcript levels for each gene expression marker within 1000+ CA1 cells. At mid‐to‐late disease timepoints, we found significant changes in specific CA1 cell types in 5xFAD mice compared to WT controls suggesting primarily susceptible as well as neuroprotective cell types to AD neurodegeneration.

**Conclusion:**

In the CA1, discrete neurons are primarily susceptible to AD neurodegeneration in the 5xFAD mouse model. Future studies will investigate the transcriptomic changes that occur within vulnerable CA1 neurons that precede death.

